# A multiplex guide RNA expression system and its efficacy for plant genome engineering

**DOI:** 10.1186/s13007-020-00580-x

**Published:** 2020-03-12

**Authors:** Youngbin Oh, Bora Lee, Hyeonjin Kim, Sang-Gyu Kim

**Affiliations:** Department of Biological Sciences, KAIST, Daejeon, 34141 Republic of Korea

**Keywords:** CRISPR-Cas9, Golden gate assembly, Multiplex gRNAs, Plant genome editing

## Abstract

**Background:**

The *Streptococcus pyogenes* CRISPR system is composed of a Cas9 endonuclease (*Sp*Cas9) and a single-stranded guide RNA (gRNA) harboring a target-specific sequence. Theoretically, *Sp*Cas9 proteins could cleave as many targeted loci as gRNAs bind in a genome.

**Results:**

We introduce a PCR-free multiple gRNA cloning system for editing plant genomes. This method consists of two steps: (1) cloning the annealed products of two single-stranded oligonucleotide fragments harboring a complimentary target-binding sequence on each strand between tRNA and gRNA scaffold sequences in a pGRNA vector; and (2) assembling tRNA-gRNA units from several pGRNA vectors with a plant binary vector containing a *Sp*Cas9 expression cassette using the Golden Gate assembly method. We validated the editing efficiency and patterns of the multiplex gRNA expression system in wild tobacco (*Nicotiana attenuata*) protoplasts and in transformed plants by performing targeted deep sequencing. Two proximal cleavages by *Sp*Cas9-gRNA largely increased the editing efficiency and induced large deletions between two cleavage sites.

**Conclusions:**

This multiplex gRNA expression system enables high-throughput production of a single binary vector and increases the efficiency of plant genome editing.

## Background

The CRISPR system derived from *Streptococcus pyogenes* consists of a CRISPR-associated protein 9 (*Sp*Cas9) endonuclease fused with a nuclear localization signal and a single-stranded guide RNA (gRNA) that transfers the *Sp*Cas9 to the target locus [1–4]. The 5′ region of the ~ 100-nt gRNA contains a ~ 20-nt target-specific sequence. By simply changing the ~ 20-nt sequence, we can quickly and easily build a tool to edit a specific gene in animals and plants. In addition, the expression of several gRNAs with *Sp*Cas9 proteins in a single cell can simultaneously edits several genes or induce the large deletion of the specific chromosome [5–13]. However, the mutation efficiency of each *Sp*Cas9-gRNA complex varies considerably, depending on the gRNA-binding loci. This variability arises because the chromatin accessibility of each complex differs depending on the target sites [14–16].

Chen et al. showed that the activity of *Francisella novicida* CAS9 (*Fn*Cas9) is increased by expressing dead Cas9 and gRNA that binds near the *Fn*Cas9 binding site [14]. When a gRNA has a 14-nt or 15-nt target binding sequence, the *Sp*Cas9-gRNA complex cannot cleave the gRNA recognition site but can bind to the target site [17, 18]. Attaching this dead complex to the vicinity of the normal gRNA-binding site increases its genome editing efficacy in rice [16]. Such results suggest that *Sp*Cas9-gRNA binding itself enhances the chromatin accessibility of other Cas9-gRNAs targeting the proximal site. This approach can be used widely to improve the efficiency of genome editing even without knowing complex chromatin structures.

Plant genome editing mostly requires a tissue culture process, which normally takes months or even a year. If multiple *Sp*Cas9-gRNA binding on proximal sites can increase the efficiency of genome editing, much effort and time can be saved. Xie et al. report that the efficiency of their two gRNA expression systems in rice is higher than that of one gRNA expression system: two gRNAs are connected by a tRNA precursor sequence and processed into individual gRNAs after transcription under the control of U6 promoter [19]. However, further research is needed to determine whether this strategy is generally applicable to gene editing in other plants.

The multiplex gRNA expressing system is convenient for generating multiple mutations in target genes. For this purpose, several toolboxes have been developed for plant genome editing. The Gibson assembly and Golden Gate assembly methods have been widely used to ligate multiple U6::gRNA cassettes and assemble them into a single plant binary vector [20, 21]. In addition, the tRNA-processing system [19], the Csy4 ribonuclease system [22] and the ribozyme system [23] have all been used to make multiplex gRNA expression constructs under a single Pol III promoter. However, the Csy4 system needs the exogenous expression of Csy4 RNase in plants and the ribozyme system showed low editing efficiency in tomato protoplasts [11]. The current version of the tRNA-processing system requires multiple PCR step to prepare constructs for editing the genomes of plants [11, 12, 19], humans [24], and mice [25]. Here, we provide a PCR-free cloning method to generate multiple tRNA-gRNA expression systems for editing plant genomes using Golden Gate assembly that requires just one week. In addition, we show that a polycistronic multi-tRNA-gRNA system increases the efficiency of gene editing in wild tobacco, *Nicotiana attenuata*.

## Results

### New system for cloning a multiplex tRNA-gRNA construct

**Fig. 1:**
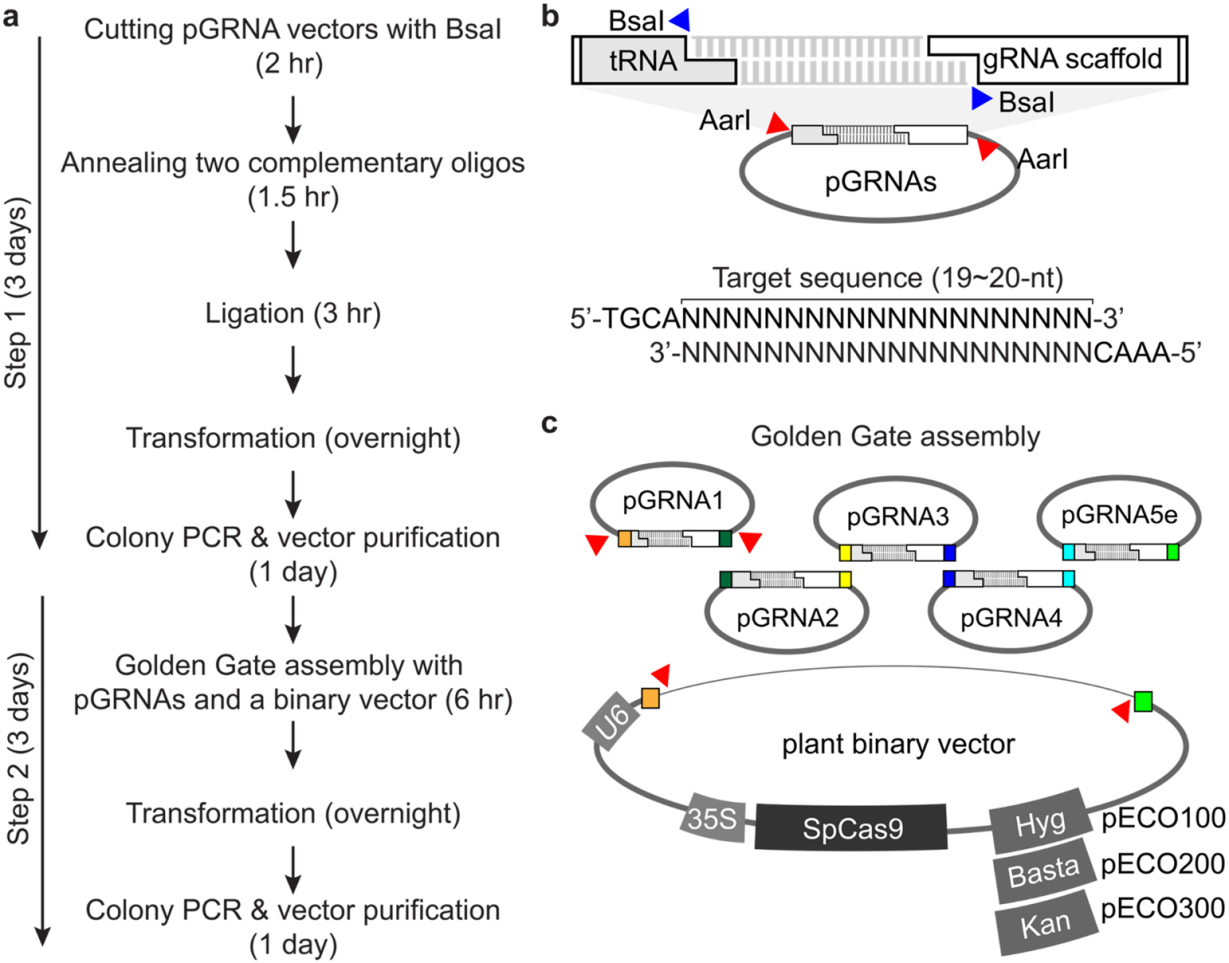
Two-step cloning system for multiplex guide RNA expression in plants. **a** Cloning procedures of multiplex guide RNAs (gRNAs). **b** The single gRNA-cloning vector, pGRNA, is designed for a PCR-free multiplex gRNA cloning method. The target-binding sequence of gRNA is prepared by annealing two complementary oligos. A pair of annealed oligos is directly cloned into the BsaI-digested pGRNA (blue triangles) between the tRNA sequence and gRNA scaffold. Two AarI sites (red triangles) are used in step 2. **c** The Golden Gate assembly for preparing a plant binary vector expressing five gRNAs under a single U6 promoter. Each tRNA-gRNA unit is excised from pGRNA by cutting the pGRNA with AarI. All tRNA-gRNA units and one of the plant binary vectors—pECO100, pECO200, or pECO300—is connected in the Golden Gate assembly mixture described in "Methods"

To express multiple gRNAs in plants, we first developed a simple, fast and PCR-free cloning method (Fig. 1). This cloning platform consists of a pre-cloned vector, pGRNA, which carries a single unit of a tRNA-gRNA scaffold, and an acceptor vector, which harbors the *Sp*Cas9-coding sequence and a selection marker (Fig. 1c). The gRNA scaffold is a fusion sequence consisting of crRNA without a 5′ end target recognition site and tracrRNA [26]. The first step is to add a target-binding sequence (19–20-nt) into the pGRNA vector by a PCR-free method (Fig. 1b). Two BsaI restriction enzyme (Type IIS) recognition sites were inserted between the tRNA sequence and the gRNA scaffold in a pGRNA vector (Fig. 1): this insertion allowed us to easily ligate a short double-stranded DNA (23–24-nt) containing the target recognition sequence into the pGRNA. To prepare the short double-stranded DNA fragment containing a target recognition sequence, we designed two complementary single-stranded oligos: one oligo starts with the 5′-TGCA-3′ sequence, followed by the target sequence and the other oligo starts with the 5′-AAAC-3′ sequence, followed by the complementary nucleotides of the target sequence (Fig. 1b). Each four additional nucleotides in the oligos was annealed to the complementary overhang sequence generated by the BsaI cut of pGRNA. We prepared each tRNA-gRNA unit within three days (no PCR step needed).

The pGRNA vector has two AarI restriction enzyme-binding sites on the outside of the tRNA-gRNA unit, and the AarI treatment produces a tRNA-gRNA unit with 4-nt overhang sequence. Each pGRNA is designed to produce specific overhang sequences that connect the tRNA-gRNA unit in the order of vector number (pGRNA1, pGRNA2, pGRNA3, pGRNA4, and pGRNA5e): tRNA-gRNA units could be sequentially ligated into a plant binary vector (acceptor vector, pECO100, pECO200, and pECO300) by using the Golden Gate assembly method (Fig. 1c). Thus, the plant binary vector with the desired multiplex gRNA combination, which we call pGG, could be easily and quickly (within a week) produced. The pGG vector was numbered according to the number of tRNA-gRNA units. For example, pGG-3 is the binary vector with three consecutive tRNA-gRNA units.

### Validation of the editing efficiency of pGG-1 and pGG-2 vectors in protoplasts

**Fig. 2:**
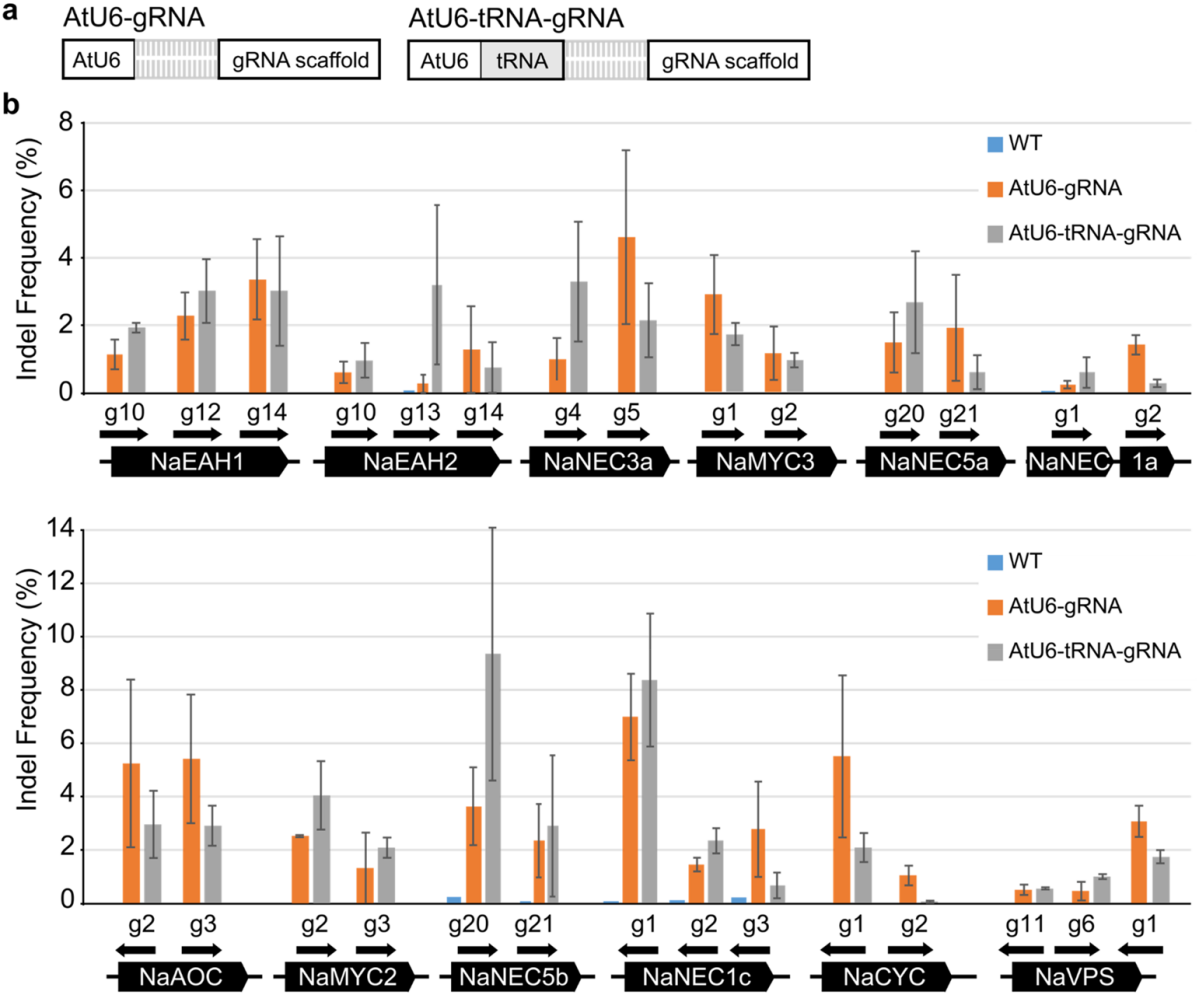
tRNA following U6 promoter does not increase the total editing efficiency of a single gRNA in wild tobacco protoplasts. **a** Structure of two types of gRNA expression systems: AtU6-gRNA and AtU6-tRNA-gRNA. **b** Comparison of indel mutation frequency between two different gRNA expression systems: AtU6-gRNA and AtU6-tRNA-gRNA. We designed 28 gRNAs to induce indel mutations in 12 *N. attenuata* genes. The coding sequences of the target genes are presented as black pentagons. Each gRNA is shown as a black arrow. Targeted deep sequencing was performed to examine indel frequency and mutation patterns at the target site. The indel frequency (%) was calculated by dividing the number of sequencing reads containing indel mutations by the number of total sequencing reads. Error bars represent standard deviation of three replicates (pools of protoplasts). AtU6, *Arabidopsis* U6-26; gRNA, guide RNA; tRNA, pre-tRNA^Gly^ gene

A part of precursor tRNA^Gly^ sequences has been used to produce multiplex gRNAs from a single polycistronic transcript driven by U6/U3 promoters. This tRNA sequence has been reported to increase the expression of gRNA in rice protoplasts, which in turn improves genome editing efficiency [19]. To determine whether the tRNA could also increase genome editing efficiency in a dicot plant, we edited twelve genes with a total of 28 gRNAs with and without the tRNA sequence in the protoplasts of wild tobacco, *N. attenuata* (Fig. 2) [27, 28]. The gRNAs were expressed under the control of either AtU6 or AtU6-tRNA (Fig. 2a). Results show that the tRNA does not increase the editing efficiency of *Sp*Cas9-gRNA complexes in *N. attenuata* (*P* = 0.56) (Fig. 2b).

**Fig. 3:**
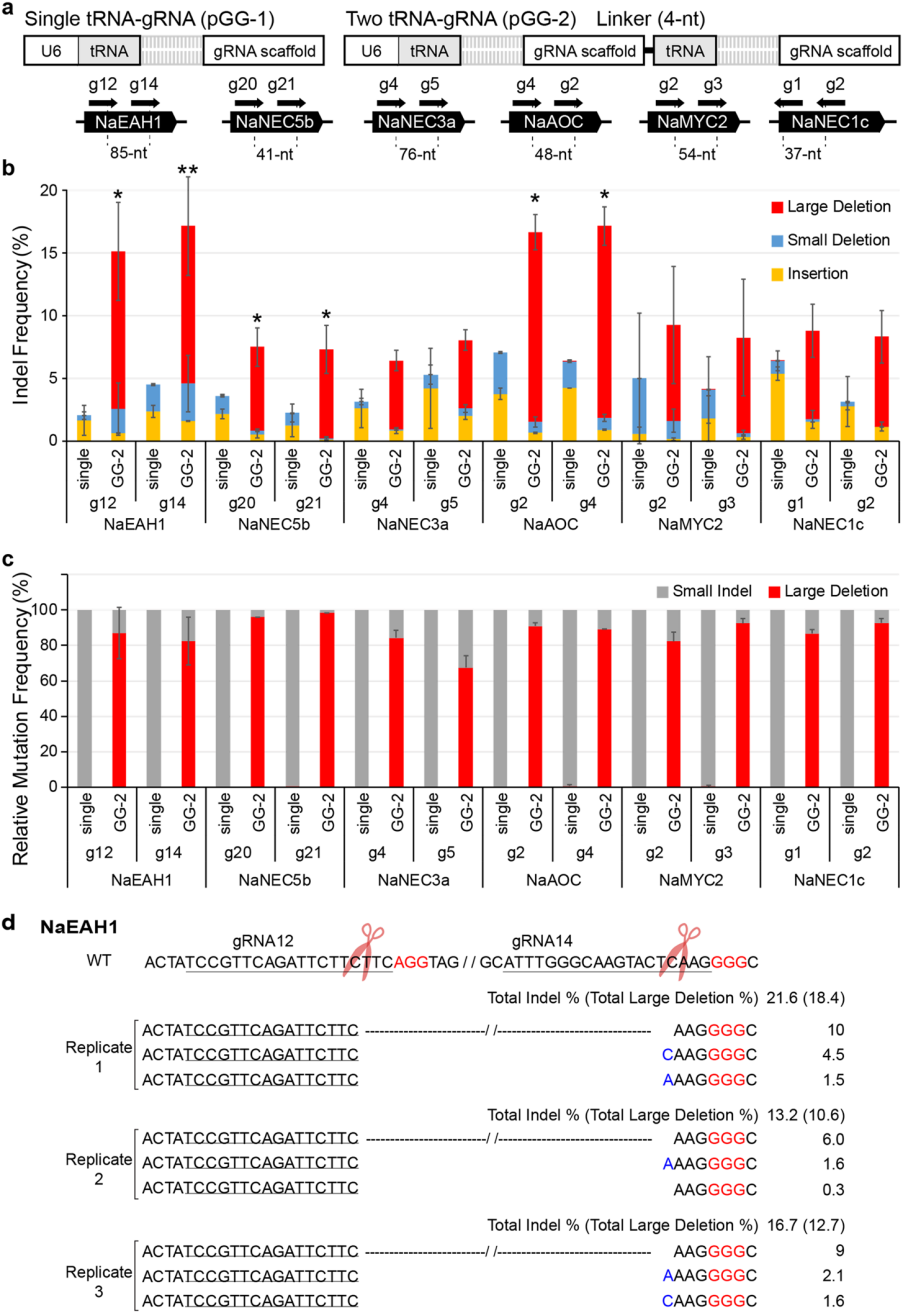
Evaluation of the multiplex guide RNA expression system for editing single gene in protoplasts. **a** Structure of a single gRNA-expressing system or two such system. A linker sequence (4-nt) is added in between the gRNA scaffold and the tRNA for Golden Gate assembly. Two gRNAs for each of six genes in *N. attenuata* were designed for mutagenesis. The coding sequences of the target genes are presented as black pentagons. Each gRNA is shown as a black arrow. Distance between two gRNA-cleavage sites are presented immediately below the black pentagons. **b** Indel frequency (%) at 12 gRNA binding sites in *N. attenuata* protoplasts. Indel mutation patterns are divided into three categories: insertion (yellow), small deletion (blue), and large deletion (red). The large deletions occur by simultaneous DNA cleavages at two adjacent gRNA-binding sites. Asterisks indicate statistically significant differences (two-tailed Student t-test, *P < 0.05, **P < 0.01). **c** Relative percentage of small indel (gray) and large deletions (red) to total mutations for each gRNA binding site. Total mutation frequency was determined by targeted deep sequencing. Error bars represent SD of three or five replicates (pools of protoplast). **d** Large deletions occur by expressing two gRNAs in the protoplasts. Wild type (WT) sequences of the *NaEAH1* gene are shown with gRNA-binding sequences (underlined) and protospacer adjacent motif (PAM) in red. In the below of WT sequence, the total indel frequency is given followed by the frequency of large deletions is in parentheses. Indels are presented in blue (insertion) and as dashes (deletion). Total Indel % is the sum of the frequency of small indels and large deletions. The DNA sequences of target locus are ranked with the large deletion frequency

We then examined whether two gRNAs targeting the proximal site of one gene increase the genome editing efficiency. We chose six target genes of *N. attenuata*—*NaEAH1, NaNEC5b*, *NaNEC3a, NaAOC*, *NaMYC2*, and *NaNEC1c*—and then designed two adjacent gRNAs to target each one (Fig. 3a). The distance between two gRNAs varied from 37- to 85-nt. The pGG vectors containing one tRNA-gRNA (pGG-1) and two tRNA-gRNA (pGG-2) units were transformed into the protoplasts, and their editing efficiency and mutation patterns were determined by targeted deep sequencing. When two gRNAs were expressed, rather than one, the editing frequency was increased at each target site: 3.0% (one gRNA) to 15% (two gRNAs, the sum of the small indel frequency induced by one gRNA and the large deletion frequency induced by two gRNAs) for NaEAH1-gRNA12 (g12), 4.5% to 17% for NaEAH1-g14, 3.6% to 8% for NaNEC5b-g20, 3.6% to 8% for NaNEC5b-g21, 3.0% to 6.4% for NaNEC3a-g4, 6.4% to 8.1% for NaNEC3a-g5, 7.1% to 16.7% for NaAOC-g2, 6.4% to 17.2% for NaAOC-g4, 5.0% to 9.3% for NaMYC2-g2, 4.5% to 8.2% for NaMYC2-g3, and 6.4% to 8.8% for NaNEC1c-g1, 4.4% to 8.3% for NaNEC1c-g2 (Fig. 3b).

We found that two proximal cleavages by *Sp*Cas9-gRNA induced large deletions between two cleavage sites (Fig. 3b). The mean frequency of large deletions was 12.6% for NaEAH1-g12 and -g14, 6.7% for NaNEC5b-g20 and 7.1% for NaNEC5b-g21, 5.4% for NaNEC3a-g4 and -g5, 15.1% for NaAOC-g2 and 15.3% for NaAOC-g4, 7.6% for NaMYC2-g2 and -g3, and 7.1% for NaNEC1c-g1 and 7.2% for NaNEC1c-g2 (Fig. 3b). In some case, the large deletion frequency of two gRNAs were slightly different because the single gRNA cleavage can also induce the large deletion by the microhomology based-NHEJ repair pathway. Although total editing frequencies (the sum of the small indel frequency and the large deletion frequency) of six pGG-2 constructs varied, the relative ratio of large deletions to total mutations was similar: the mean frequency of the relative ratio of the large deletions was ~ 85% for NaEAH1-g12-g14, ~ 97% for NaNEC5b-g20-g21, ~ 76% for NaNEC3a-g4-g5, ~ 90% for NaAOC-g2-g4, ~ 87% for NaMYC2-g2-g3, and ~ 90% for NaNEC1c-g1-g2 (Fig. 3c). The precise large deletion occurred by rejoining the blunt end of two cleaved sites at three nucleotides upstream of the protospacer adjacent motif (PAM) sequence without any insertion or deletion of nucleotides: the mean frequencies of the relative ratio of precise large deletion to total large deletions were ~ 60% for NaEAH1-g12-g14, ~ 38% for NaNEC5b-g20-g21, ~ 84% for NaNEC3a-g4-g5, ~ 95% for NaAOC-g2-g4, ~ 28% for NaMYC2-g2-g3, and ~ 63% for NaNEC1c-g1-g2 (Fig. 3d and Additional file 1). The next abundant mutation patterns were revealed by the large deletions with one nucleotide insertion or deletion at each cleaved site. For instance, either the C or A nucleotide was added at the NaEAH1-g14-cleaved site (Fig. 3d); A was added at the NaNEC5b-g21-cleaved site or GG was removed at the NaNEC5b-g20-cleaved site; three different nucleotides—A, T, or C—were added at the NaNEC3a-g5-cleaved site or GA was removed at the NaNEC3a-g4-cleaved site; A was removed at the NaAOC-g4-cleaved site; A was added at the NaMYC2-g3-cleaved site or one or four nucleotides was removed at the NaMYC2-g2-cleaved site; and T was added at the NaNEC1c-g1-cleaved site or several nucleotides were removed at the NaNEC1c-g2-cleaved site (Additional file 1).

### Genome editing with three (pGG-3) and four gRNAs (pGG-4) in protoplasts and in planta

**Fig. 4:**
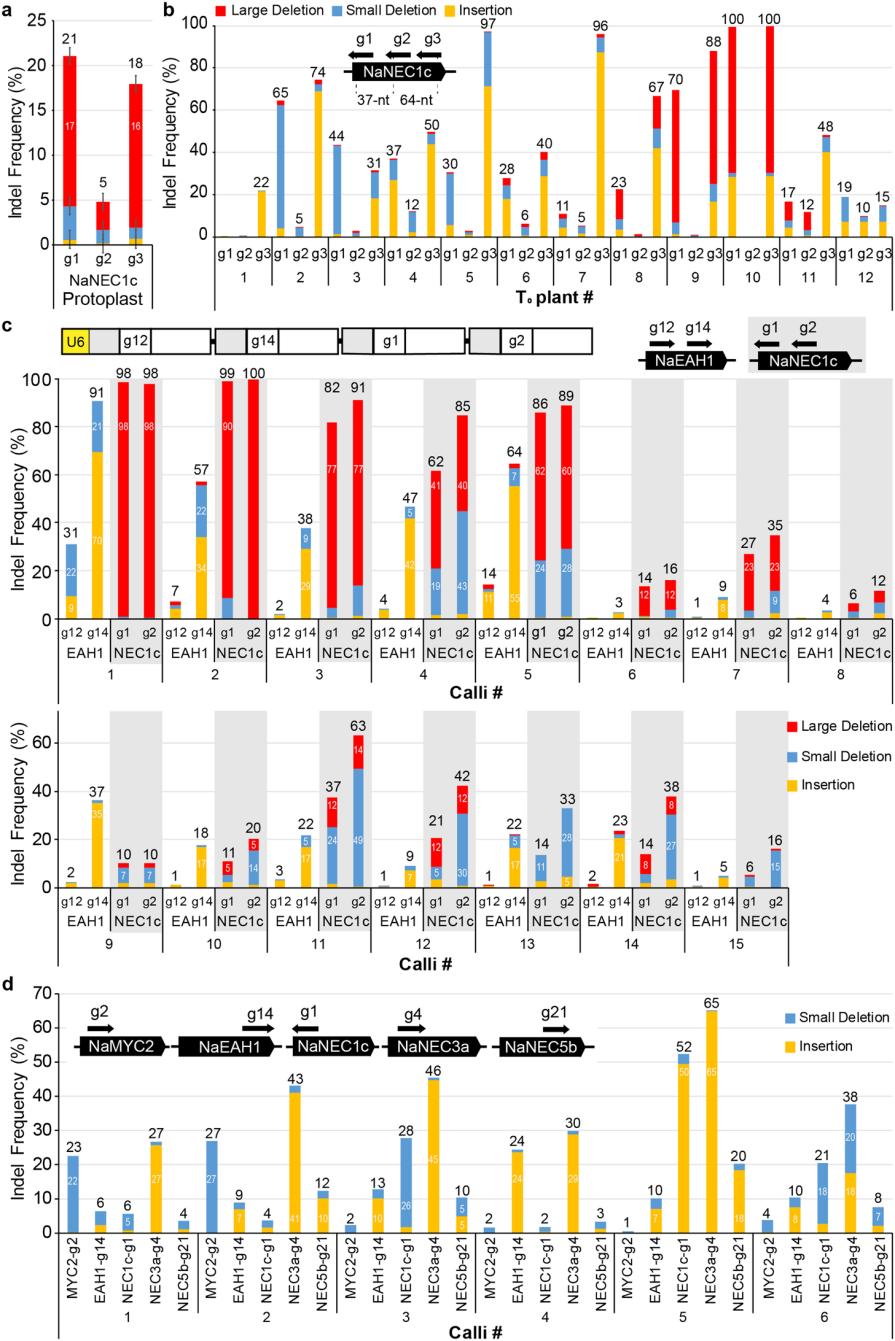
Validating the multiplex guide RNA expression system for generating genome-edited plants. **a** Indel frequency (%) at three gRNA binding sites in the *NaNEC1c* gene in protoplasts harboring the pGG-3 vectors. Large deletions are individually calculated at NaNEC1c-g1, -g2, or -g3- binding site. For instance, the large deletion at the NaNEC1c-g1-binding site is calculated by the sum of large deletion occurred between the target sites of -g1 and -g2 and large deletion occurred between the target sites of -g1 and -g3. Error bars represent SD of three replicates (pools of protoplasts). The colors used in the graph represent the different mutation patterns described in **b**. **b** Indel frequency (%) in *N. attenuata* T_0_ plants harboring the pGG-3 vectors. **c** Indel frequency (%) in *N. attenuata* transformed calli harboring the pGG-4 is calculated by the sum of small indel frequency and large deletion frequency at each gRNA-binding site. Schematic maps of gRNA12, gRNA14, gRNA1, and gRNA2 expression vector. gRNA12 and gRNA14 bind to the first exon of *NaEAH1* and gRNA1 and gRNA2 bind to the first exon of *NaNEC1c*. Large deletions occurred between the target sites of gRNA12 and 14, and between the target sites of gRNA1 and 2. **d** Indel frequency (%) in *N. attenuata* calli harboring the pGG-5 vectors. *Agrobacterium* was used to transform *N. attenuata*. Total mutation frequency was calculated by targeted deep sequencing

Furthermore, we examined the editing efficiency of pGG-3 constructs in protoplasts. In Fig. 3b, we examined the efficiency with which two guide RNAs edit the *NaNEC1c* gene. The third gRNA, NaNEC1c-g3 was designed to cleave the double-stranded DNA at 64-nt apart from the NaNEC1c-g2 cleavage site (Fig. 4a, b). We then examined the mutation patterns induced by simultaneously expressing three gRNAs binding on the proximal target sites. The total mutation frequency of NaNEC1c-g1-g2-g3-transformed protoplasts was 25.7% including small indels (4.3% for NaNEC1c-g1, 1.6% for NaNEC1c-g2, and 1.9% for NaNEC1c-g3) and large deletions (14.8% for NaNEC1c-g1 and -g3, 1.9% for NaNEC1c-g1 and -g2, 1.2% for NaNEC1c-g2 and -g3) (Fig. 4a).

We next tested whether the pGG system could effectively edit target genes in planta and induce the similar mutation patterns observed in the protoplasts. The pGG-3 vector carrying NaNEC1c-g1-g2-g3 was delivered into *N. attenuata* hypocotyl explants using *Agrobacterium*-mediated transformation [29] and whole plants were regenerated on the selection media. Gene editing was observed for at least one binding site of three gRNAs in 21 T_0_ lines among 24 T_0_ transformants (87.5%, Fig. 4b and Additional file 2). As shown in the protoplasts, the editing frequency at the NaNEC1c-g2-binding site was lower than the editing frequency at the NaNEC1c-g1 and -g3-binding sites (Fig. 4a, b). Some T_0_ lines (T_0_-8, -9, -10) had large deletions at the target site: the major mutation pattern of the large deletion occurred when the blunt ends of two cleaved sites were rejoined at three nucleotides upstream of the PAM sequence of NaNEC1c-g3 and NaNEC1c-g1 with T insertion (Fig. 4b and Additional file 3b). However, unlike the results with the protoplasts, the results with several T_0_ transformants (T_0_-1, 2, 3, 4, 5, 6, 7, 12) had small indel mutations (Fig. 4b). Major small indel patterns in transformed plants exhibited an A or T insertion at the three nucleotides upstream of the PAM sequence of NaNEC1c-g3-binding site (Additional file 4).

To validate the heritability of the targeted mutation induced by our system, we collected the seeds from the T_0_-2 transgenic plant harboring a NaNEC1c-g1-g2-g3 construct and germinated these T_1_ seeds. The major indel patterns of T_1_-2–9 line was a single-nucleotide (T) insertion at the gRNA3-cleaved site and two nucleotide deletion at the gRNA1-cleaved site, which is the major mutation pattern of T_0_-2 plant. Interestingly, T_1_-2–20 line contains a single-nucleotide (A) insertion at the gRNA3-cleaved site and the large deletion between the gRNA2- and gRNA3-cleaved sites, which is the minor mutation patterns of T_0_-2 plant (Additional file 5).

We also confirmed that the pGG-4 vector carrying four gRNAs can successfully edit two genes in plants: g12 and g14 for targeting *NaEAH1*, and g1 and g2 for targeting *NaNEC1c* (Fig. 4c). Genomic DNA was extracted from the transformed calli grown in the selection media and the mutation frequency of each callus was measured by the sum of small indel frequency and large deletion frequency. At least one gene was edited from 15 out of 16 calli (94%). Furthermore, the four-gRNA expression with *Sp*Cas9 successfully generated mutations both on *NaEAH1* and *NaNEC1c* (more than 50% mutation frequency) in the calli 1, 2, and 5. The mutation patterns of *NaEAH1* in the protoplasts and the calli were quite different: the dominant mutation pattern in the calli (Fig. 4c and Additional file 6) was insertion mutations, whereas the dominant mutation pattern in the protoplast was the large deletions (Fig. 3b). NaNEC1c-g1-g2 induced large deletions in both protoplasts (Fig. 3b) and the calli (Fig. 4c and Additional file 6). In callus-1, -2, and -3, T was inserted at the NaNEC1c-g1 cleaved site (Additional file 6), which was also observed in the NaNEC1c-g1-g2 transfected protoplasts (Additional file 1).

Finally, we validated the editing efficiency of the pGG-5 vector in plants: NaMYC2-g2, NaEAH1-g14, NaNEC1c-g1, NaNEC3a-g4, and NaNEC5b-g21 were cloned into the pECO100 (Fig. 4d). After the *Agrobacterium*-mediated transformation, we extracted genomic DNA from the transformed calli and measured the indel frequency of each gRNA. The indel frequencies of each gRNA varied in the different calli (Fig. 4d). For instance, the indel frequency at the NaMYC2-g2-cleaved site was 22.5%, 26.9%, 2.3%, 1.7%, 0.5%, and 3.7% in the calli-1, -2, -3, -4, -5, and -6, respectively. The indel frequency at the NaNEC3a-g4-cleaved site was 26.7%, 43.1%, 45.5%, 29.8%, 65.2%, and 37.6% in the calli-1, -2, -3, -4, -5, and -6, respectively. While the indel frequency of five gRNAs in a single callus differed each other considerably, the five-gRNA expression system successfully induced the targeted mutation in a single callus (Fig. 4d).

## Discussion

In this study, we tested the activity of our multiple gRNA expression system in wild tobacco protoplasts and also in transformed plants. To increase the reliability of the protoplast assay, three protoplast transfections were carried out independently, and the mutation frequency and patterns of each transfection were analyzed by targeted deep sequencing. We designed 29 gRNAs for targeting 12 genes and delivered a binary vector harboring several combinations of gRNAs in plant cells. Measurements of editing efficiency with targeted deep sequencing clearly show that the expression of two nearby gRNAs generally increases the editing efficiency more than the expression of a single gRNA in wild tobacco protoplasts (Fig. 3). This result suggests that binding of *Sp*Cas9-gRNA on DNA might change the chromatin structure near the binding site and increase the accessibility of *Sp*Cas9 and the other gRNA to the target locus. Thus, our multiple gRNA expression system will be useful for improving the activity of base editing with CRISPR-mediated base editing tools [30]. The distances between two nearby gRNAs used in this study were 50–100-nt for each gene. Future investigation should determine the effect of the distance between two gRNAs on editing efficiency.

Unlike the previous report [19], we found that the tRNA sequence itself did not affect the editing efficiency of *N. attenuata* (Fig. 2). We used the glycine tRNA from a monocot plant, rice used in the previous study [19]. Although the rice glycine tRNA works well for expressing multiplex gRNA in *N. attenuata*, the rice tRNA seems to play no role for enhancing transcription in *N. attenuata*. In addition, the tRNA system enables to produce at least one to five gRNAs in a single cell.

We compared the mutation patterns induced in protoplasts with the mutation patterns in transformant tissues. As shown in Figs. 3 and 4a, a large deletion is the major mutation pattern when two nearby gRNAs target the *NaEAH1* and *NaNEC1c* in the protoplasts. However, in some transgenic plants and calli, we can find different types of mutation patterns. We hypothesize that patterns reflect the difference of the chromatin structure between protoplasts and hypocotyl tissues that we used for *Agrobacterium*-mediated transformation [31].

## Conclusions

In conclusion, we developed a user-friendly toolbox to prepare a plant binary vector expressing multiple gRNAs for genome editing. We also validated the genome-editing efficiency of our vector system in wild tobacco protoplasts and transgenic plants, and showed how to increase the editing efficiency by expressing two nearby gRNAs. This toolbox enables high-throughput production of a single binary vector for editing multiple genomic sites in plants.

## Methods

### Vector construction and guide RNA design

The pGRNA vectors were generated from the All in One™ vector (BIOFACT, Daejeon, Korea) with some modifications: the ccdB gene was replaced with the multiple cloning site; 77-nt tRNA sequence [19] was synthesized by the manufacturer (Macrogen, Seoul, Korea); tRNA sequence and gRNA scaffold were added into the multiple cloning site; the BsaI binding site in the All in One™ vector was removed and two new BsaI binding sites were added between the tRNA and gRNA scaffold; and two AarI binding sites were added in pGRNA0 vectors (Additional file 7). pGRNA vectors were generated from the pGRNA0 vector. Each pGRNA has the unique linker sequence used in the Golden Gate assembly. The plant binary vectors (pECO100, pECO200, and pECO300) were modified from pHAtC [32]. The sequences of all pGRNA vectors are in Additional file 7, and all constructs with the sequence information will be deposited in AddGene. The primers used in vector constructions are listed in Additional file 8.

The target binding sequence (19–20-nt) in gRNA was synthesized by the manufacturer (Macrogen) and cloned into the BasI-cut pGRNAs as previously described [32] with minor modifications. The oligos were annealed using the T4 ligation buffer (NEB, Ipswich, MA, USA) in a thermocycler (95 °C for 5 min, 95 °C to 25 °C with a − 1 °C/min, and 10 °C). pGRNA vectors were digested with BsaI (NEB) and used for the ligation reaction. The digested pGRNA vector was ligated together with the annealed oligos using T4 DNA ligase (NEB). The ligation mixture was incubated at RT (room temperature) for 2 h.

The Golden Gate assembly was performed following the protocol described in Andreou and Nakayama [33]. In briefly, the Golden Gate assembly was carried out in 20 μL reaction comprised of 50 ng of acceptor vector (pECO100) and 24 ng of pGRNA vectors (pGRNA1 and pGRNA2e for expressing two gRNAs; pGRNA1, pGRNA2, and pGRNA3e for three gRNAs; pGRNA1, pGRNA2, pGRNA3 and pGRNA4e for four gRNAs; pGRNA1, pGRNA2, pGRNA3, pGRNA4 and pGRNA5e for five gRNAs) in addition to 2 μL of 1 mg/mL BSA (NEB), 2 μL of T4 DNA ligase buffer (NEB), 1 μL of AarI (Thermo Fisher Scientific, Waltham, MA, USA), and 0.4 μL of 50× oligos (Thermo Fisher Scientific) in the following thermocycler conditions: 40 cycles of (37 °C for 5 min, 16 °C for 10 min) followed by 5 min at 37 °C and 5 min at 80 °C for enzyme inactivation.

The target sequences of gRNAs were designed using the Cas-Designer program, which is available at CRISPR RGEN Tools (https://www.rgenome.net/) [27, 28], and primers used in this study were synthesized by Bioneer (Daejeon, Korea) and Macrogen. To edit one gene, we first designed several gRNAs recommended by the CRISPR RGEN tools and gave the arbitrary number to each gRNA. And then we chose two or more gRNAs based on the experimental purpose or the rule of thumb proposed in previous studies [34, 35]. There is no specific rule for numbering of gRNA. Genome sequence information for *N. attenuata* is available in the *Nicotiana attenuata* Data Hub (https://nadh.ice.mpg.de/NaDH/).

### *Agrobacterium*-mediated wild tobacco transformation

The *N. attenuata* Utah wild-type seeds were originally collected from plants growing in southwestern Utah in the USA and were a gift from the Department of Molecular Ecology at the Max Plank Institute for Chemical Ecology. Seeds were sterilized and germinated following the protocol described in Krugel et al*.* [29]. The plants were grown under long day conditions (16 h light/8 h dark) at 25 °C with ± 1 °C in a plant growth chamber (JSR, Daejeon, Korea). The binary vectors were transformed into the *Agrobacterium tumefaciens* strain LBA4404 by the thaw-freeze method. The hypocotyl tissues were used for tissue culture and transformation [29].

### Protoplast isolation and transfection

Protoplasts were isolated as previously described [36] with minor modifications. Four-week-old *N. attenuata* leaves were digested with enzymes: 1% viscozyme (Novozymes, Copenhagen, Denmark), 0.25% celluclast (Novozymes), 0.25% pectin EX (Novozymes), 0.2 M potassium dihydrogen phosphate (Duchefa, haarlem, The Netherlands), 1 M potassium nitrate (Duchefa), 1 M magnesium sulphate heptahydrate (Duchefa), 1 mM potassium iodide (Duchefa), 0.1 mM cupric sulphate pentahydrate (Duchefa), 10 mM calcium chloride dehydrate (Sigma-Aldrich, Saint Louis, USA), 0.5 M mannitol (Sigma-Aldrich), 5 mM MES (MBcell, Seoul, Korea) [pH 5.8], for 1.5 h at RT in the dark. Subsequently, protoplasts were filtered through a 100 μM cell strainer and washed with W5 solution (4 mM sodium chloride, 125 mM calcium chloride dihydrate, 5 mM potassium chloride, 5 mM d-glucose monohydrate, 1.5 mM MES, pH 5.6). Protoplasts were then applied to a 21% sucrose gradient followed by centrifugation at 50 g for 5 min. The intact protoplasts were re-suspended in W5 solution and stabilized for 1 h at 4 °C before PEG-calcium transfection. PEG-calcium DNA transfections were performed following previously described protocols [36, 37]. Briefly, 2 × 10^5^ protoplast cells were transfected with 30 μg of plasmids. Protoplasts re-suspended in MMG solution (4 mM MES, 0.4 M mannitol, 1.5 mM magnesium chloride hexahydrate, pH 5.7) were mixed with plasmids and freshly made PEG solution, and then incubated at RT for 20 min. After incubation, protoplasts were washed three times with an equal volume of W5 solution. The washing step was done by slowly rolling the tube. Protoplasts were pelleted by centrifugation at 50 g for 5 min and re-suspended in WI solution (0.5 M mannitol, 4 mM MES, 20 mM potassium chloride, pH 5.7). Finally, the protoplast cells were cultured under dark conditions at 25 °C for 72 h.

### Targeted deep sequencing and data analysis

The genomic DNA for targeted deep sequencing analysis was extracted from protoplasts, calli, or randomly selected leaves in T_0_ plants using HiGene Genomic DNA prep kit (BIOFACT). The target sites were amplified from genomic DNA using gene-specific primers. Indices and sequencing adaptors were attached by additional PCR steps. High-throughput sequencing was performed using Illumina Miseq (v2, 300-cycle, San Diego, CA, USA). The mutation frequency and patterns were analyzed using the Cas-Analyzer program implemented in CRISPR RGEN Tools (https://www.rgenome.net/). The indel frequency (%) was calculated by dividing the number of sequencing reads containing indel mutations by the number of total sequencing reads. The large deletion frequency (%) was calculated by dividing the number of sequencing reads containing large deletion by the number of total sequencing reads. The total mutation (indel) frequency induced by more than two gRNAs was calculated by the sum of the small indel frequency induced by single gRNA and the large deletion frequency induced by the two gRNAs. The relative percentage of small indels or large deletions to total mutations was calculated by dividing the number of sequencing reads containing small indels or large deletions by the number of total mutation reads. The mean frequency of the relative ratio of precise large deletion to total large deletions was calculated by using the read number of large deletions (Fig. 3d). For instance, the read number of total large deletions for NaEAH1-g12-g14 was 1746 and the read number of precise large deletions was 949 in the first replicate. Thus, the relative ratio of the precise large deletion to total large deletions was 54.4% in the first replicate. The relative ratios in second and third replicates were 55.2% and 70.9%, respectively. The mean frequency of the relative ratio of precise large deletion to total large deletions were ~ 60% for NaEAH1-g12-g14. Large deletions occurred in the three gRNA-transformed protoplasts and T_0_ plants were individually calculated at each gRNA-binding site. For instance, the large deletion at the NaNEC1c-g1-binding site was calculated by the sum of large deletion occurred between the target sites of -g1 and -g2 and large deletion occurred between the target sites of -g1 and -g3 (Fig. 4a, b). Similarly, the large deletion at the NaNEC1c-g2-binding site was calculated by the sum of large deletion occurred between the target sites of -g1 and -g2 and large deletion occurred between the target sites of -g2 and -g3 (Fig. 4a, b).

## Supplementary information

**Additional file 1.** Large deletions induced by pGG-2 in protoplasts. The sequences of representative large deletion products in protoplasts. Wild type (WT) sequences of NaNEC5b (a), NaNEC3a (b), NaAOC (c), NaMYC2 (d), and NaNEC1c (e) are shown with guide sequences (underlined) and protospacer adjacent motif (PAM) in red. Indels are presented in blue (insertion) and as dashes (deletion). Total Indel % is the sum of the frequency of small indels and large deletions. The DNA sequences of target locus are ranked with the large deletion frequency.

**Additional file 2.** Indel frequency (%) in *N. attenuata* T_0_ plants harboring the pGG-3 vectors. Indel frequency (%) in *N. attenuata* T_0_ plants was calculated by the sum of small indel frequency and large deletion frequency at each gRNA-binding site.

**Additional file 3.** Large deletions induced by pGG-3 in protoplasts and T_0_ plants. The sequences of representative large deletion products in protoplasts (a) and T_0_ plants (b). Wild type (WT) sequences of NaNEC1c are shown with gRNA-binding sequences (underlined) and protospacer adjacent motif (PAM) in red. Indels are presented in blue (insertion) and as dashes (deletion). Total Indel % is the sum of the frequency of small indels and large deletions. The DNA sequences of target locus are ranked with the large deletion frequency.

**Additional file 4.** Small indels induced by pGG-3 in T_0_ plants. Small indels observed in T_0_ plants. Wild type (WT) sequences of NaNEC1c are shown with gRNA-binding sequences (underlined) and protospacer adjacent motif (PAM) in red. The DNA sequences of target locus are ranked with the mutation frequency. Indels are presented in blue (insertion) and as dashes (deletion). Total Indel % is the sum of the frequency of small indels and large deletions. The DNA sequences of target locus are ranked with the indel frequency.

**Additional file 5.** Heritability of targeted mutations in *N. attenuata*. (a) Indel mutations observed in T_0_-2 plants. (b) Sanger sequencing results from two progenies of T_0_-2 plants. T_1_-2–9 lines show the small deletion at the gRNA1-cleaved site and the single-nucleotide insertion at the gRNA3-claved site. T_1_-2–20 lines show the large deletion between the gRNA1- and gRNA2-cleaved sites and the single-nucleotide insertion at the gRNA3-claved site. Total Indel % is the sum of the frequency of small indels and large deletions. The DNA sequences of target locus are ranked with the indel frequency.

**Additional file 6.** Mutation patterns induced by pGG-4 in callus. Large deletions and small indels observed in calli -1, -2, -3, -4, and –5. Wild type (WT) sequences of NaNEC1c and NaEAH1 are shown with guide sequences (underlined) and protospacer adjacent motif (PAM) in red. The DNA sequences of target locus are ranked with the mutation frequency. Indels are presented in blue (insertion) and as dashes (deletion).

**Additional file 7.** Full sequences of pGRNA vectors.

**Additional file 8.** List of primers used in this study.

## Data Availability

All vectors—pECO100, pECO200, pECO300, pGRNA1, pGRNA2, pGRNA3, pGRNA4, pGRNA2e, pGRNA3e, pGRNA4e, and pGRNA5e—used in this study will be deposited in AddGene. All data generated during this study are included in this published article and its additional files.

## Abbreviations

AtU6: *Arabidopsis* u6-26
crRNA: CRISPR RNA
*Fn*Cas9: *Francisella novicida* CAS9
gRNA: Guide RNA
Indel: Insertion and deletion mutation
NaAOC: Allene oxide cyclase
NaCYC: Transcription factor CYCLOIDEA
NaEAH1: Premnaspirodiene oxygenase-like
NaEAH2: Premnaspirodiene oxygenase-like
NaMYC2: Transcription factor MYC2-like
NaMYC3: Transcription factor MYC2-like
NaNEC1a: Nectarin-1-like 1a
NaNEC1c: Nectarin-1-like 1c
NaNEC3a: Nectarin-3-like
NaNEC5a: Cannabidiolic acid synthase-like
NaNEC5b: Cannabidiolic acid synthase-like
NaVPS: Vetispiradiene synthase 3-like
PAM: Protospacer adjacent motif
pGG: Plant binary vector with the desired multiplex gRNA combination
pGRNA: Pre-cloned vector with single gRNA
*Sp*Cas9: *Streptococcus pyogenes* CAS9
tracrRNA: Trans-acting crRNA
tRNA: Pre-tRNA^Gly^ gene
