# Supplementary material for: A multiplex guide RNA expression system and its efficacy for plant genome engineering
Source: Plant Methods. 2020 Mar 12;16:37. doi: 10.1186/s13007-020-00580-x (PMC7069183; doi:10.1186/s13007-020-00580-x)
Supplement: Supplementary file 1 — Additional file 1. Large deletions induced by pGG-2 in protoplasts. The sequences of representative large deletion products in protoplasts. Wild type (WT) sequences of NaNEC5b (a), NaNEC3a (b), NaAOC (c), NaMYC2 (d), and NaNEC1c (e) are shown with guide sequences (underlined) and protospacer adjacent motif (PAM) in red. Indels are presented in blue (insertion) and as dashes (deletion). Total Indel % is the sum of the frequency of small indels and large deletions. The DNA sequences of target locus are ranked with the large deletion frequency. [file 13007_2020_580_MOESM1_ESM.pdf]

Additional file 1

a NaNEC5b

gRNA20 gRNA21

WT TTTTGGGCTATAAGAGGAGGTGGAGGCAA / / TTCTTGCATGGAAAATCAAATTGGC

		Total Indel % (Total Large Deletion %)	10.1 (8.7)
Replicate 1	TTTTGGGCTATAAGAGGAGG----------------------/ /--------------------------------	AAATTGGC	5.1
	TTTTGGGCTATAAGAGGAGG	AATTGGC	2.9
	TTTTGGGCTATAAGAGGA - -	AATTGGC	0.3
		Total Indel % (Total Large Deletion %)	5.6 (5.1)
Replicate 2	TTTTGGGCTATAAGAGGAGG ---------------------/ /--------------------------------	AAATTGGC	2.6
	TTTTGGGCTATAAGAGGAGG	AATTGGC	1.9
		Total Indel % (Total Large Deletion %)	6.9 (6.2)
Replicate 3	TTTTGGGCTATAAGAGGAGG ---------------------/ /--------------------------------	AATTGGC	2.7
	TTTTGGGCTATAAGAGGAGG	AAATTGGC	2.5

b NaNEC3a

gRNA4 gRNA5

WT AATAGTTGAAGATACAATGAGGATGGCAG / / TCTTGCAAGATCTGGAGAAGTTGGT

		Total Indel % (Total Large Deletion %)	6.9 (5.9)
Replicate 1	AATAGTTGAAGATACAATGA ---------------------/ /--------------------------------	AGTTGGT	4.5
	AATAGTTGAAGATACAATGA	TAGTTGGT	0.5
	AATAGTTGAAGATACAATGA	AAGTTGGT	0.5
		Total Indel % (Total Large Deletion %)	5.5 (4.3)
Replicate 2	AATAGTTGAAGATACAATGA ---------------------/ /--------------------------------	AGTTGGT	3.8
	AATAGTTGAAGATACAATGA	AAGTTGGT	0.3
		Total Indel % (Total Large Deletion %)	6.9 (6.1)
Replicate 3	AATAGTTGAAGATACAATGA ---------------------/ /--------------------------------	AGTTGGT	5.3
	AATAGTTGAAGATACAAT - -	AGTTGGT	0.2
	AATAGTTGAAGATACAATGA	CAGTTGGT	0.2

c NaAOC

gRNA4 gRNA2

WT TACGAGCTCAATGAACGTGACCGTGGTAG / / CCAAAAGACTGTCAATTCCCTTGGA

		Total Indel % (Total Large Deletion %)	14.8 (13.7)
Replicate 1	TACGAGCTCAATGAACGTGA ---------------------/ /--------------------------------	CCTTGGA	12.8
	TACGAGCTCAATGAACGTG -	CCTTGGA	0.5
		Total Indel % (Total Large Deletion %)	18.5 (16.8)
Replicate 2	TACGAGCTCAATGAACGTGA ---------------------/ /--------------------------------	CCTTGGA	16.3
	TACGAGCTCAATGAACGTG -	CCTTGGA	0.4

d NaMYC2

gRNA2 gRNA3

WT TTGTATTCCTTCAGCAAACGGCGTGGTTG / / AGAGTTCTGATCTCATGAACAAGGT

		Total Indel % (Total Large Deletion %)	6.4 (5.6)
Replicate 1	TTGTATTCCTTCAGCAAAC - ---------------------/ /--------------------------------	ACAAGGT	2.2
	TTGTATTCCTTCAGCAAACG	ACAAGGT	2
		Total Indel % (Total Large Deletion %)	2 (1.7)
Replicate 2	TTGTATTCCTTCAGCAAACG ---------------------/ /--------------------------------	ACAAGGT	1
	TTGTATTCCTTCAGCAAAC -	ACAAGGT	0.1
		Total Indel % (Total Large Deletion %)	18.6 (16)
Replicate 3	TTGTATTCCTTCAGCAAACG ---------------------/ /--------------------------------	ACAAGGT	4
	TTGTATTCCTTCAGCAAAC -	ACAAGGT	3.5
	TTGTATTCCTTCAGCAAACG	AACAAGGT	2.6
		Total Indel % (Total Large Deletion %)	8.7 (7.3)
Replicate 4	TTGTATTCCTTCAGCAAACG ---------------------/ /--------------------------------	ACAAGGT	2.4
	TTGTATTCCTTCAGCAAACG	AACAAGGT	1.1
	TTGTATTCCTTCAGCAAAC -	ACAAGGT	1
		Total Indel % (Total Large Deletion %)	10.6 (9.4)
Replicate 5	TTGTATTCCTTCAGCAAACG ---------------------/ /--------------------------------	ACAAGGT	2.6
	TTGTATTCCTTCAGCAAAC -	ACAAGGT	1.3
	TTGTATTCCTTCAGCA - - - -	ACAAGGT	0.8

e NaNEC1c

gRNA2 gRNA1

WT CTAGGATCAAAGGAAGAACCAAAAGGAAA / / AACAATGACTAAGATAATTGTGAGGC

		Total Indel % (Total Large Deletion %)	5.3 (4.4)
Replicate 1	CTAGGATCAAAGGAAGAACC ---------------------/ /--------------------------------	GTGAGGC	2.2
	CTAGGATCAAAGGAAGAACC	TGTGAGGC	0.6
	CTAGGATCAAAGGA - - - - - -	TGTGAGGC	0.5
		Total Indel % (Total Large Deletion %)	11.3 (9.6)
Replicate 2	CTAGGATCAAAGGAAGAACC ---------------------/ /--------------------------------	GTGAGGC	7.7
	CTAGGATCAAAGGAAGAAC -	TGTGAGGC	1.3
	CTAGGATCAAAG - - - - - - - -	TGTGAGGC	0.3
		Total Indel % (Total Large Deletion %)	9.7 (7.2)
Replicate 3	CTAGGATCAAAGGAAGAACC ---------------------/ /--------------------------------	GTGAGGC	4.3
	CTAGGATCAAAGGAAGAAC -	TGTGAGGC	1
