# Supplementary figures and images for: A multiplex guide RNA expression system and its efficacy for plant genome engineering
Source: Plant Methods. 2020 Mar 12;16:37. doi: 10.1186/s13007-020-00580-x (PMC7069183; doi:10.1186/s13007-020-00580-x)

Additional file 2

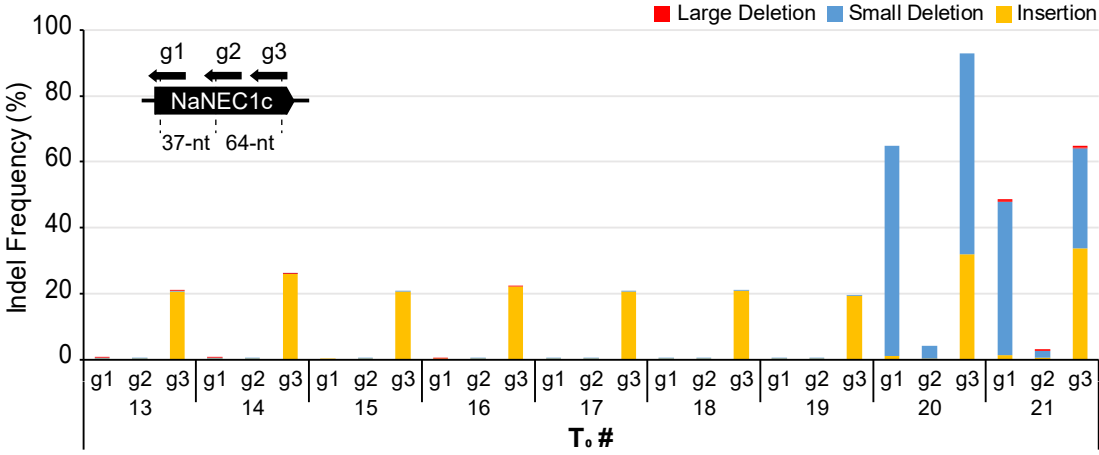

Supplement: Supplementary file 2 — Additional file 2. Indel frequency (%) in N. attenuata T0 plants harboring the pGG-3 vectors. Indel frequency (%) in N. attenuata T0 plants was calculated by the sum of small indel frequency and large deletion frequency at each gRNA-binding site. [file 13007_2020_580_MOESM2_ESM.pdf]
