# Supplementary material for: A multiplex guide RNA expression system and its efficacy for plant genome engineering
Source: Plant Methods. 2020 Mar 12;16:37. doi: 10.1186/s13007-020-00580-x (PMC7069183; doi:10.1186/s13007-020-00580-x)
Supplement: Supplementary file 3 — Additional file 3. Large deletions induced by pGG-3 in protoplasts and T0 plants. The sequences of representative large deletion products in protoplasts (a) and T0 plants (b). Wild type (WT) sequences of NaNEC1c are shown with gRNA-binding sequences (underlined) and protospacer adjacent motif (PAM) in red. Indels are presented in blue (insertion) and as dashes (deletion). Total Indel % is the sum of the frequency of small indels and large deletions. The DNA sequences of target locus are ranked with the large deletion frequency. [file 13007_2020_580_MOESM3_ESM.pdf]

Additional file 3

NaNEC1c

gRNA3 gRNA2 gRNA1

WT CTGCAGACTTGGAGT / / AAGGAAGAACCAAAAGGAAA / / TAAGATAATTGTGAGGC

a

Total Indel % (Total Large Deletion %) 19.6 (16.7)

Replicate 1	CTGCAG --/ /---------------------------------- TGTGAGGC	10
	CTGCAG --/ /---------------------------------- GTGAGGC	1.0
	CTGCAG ----------------/ /------------------ CAAAAGGAAA / / TAAGATAATTGTGAGGC	0.9
	CTGCAGACTTGGAGT / / AAGGAAGAACC -----------------/ /---------------- GTGAGGC	0.8

Total Indel % (Total Large Deletion %) 25.8 (21.5)

Replicate 2	CTGCAG --/ /---------------------------------- TGTGAGGC	16.8
	CTGCAG --/ /---------------------------------- GTGAGGC	1.7
	CTGCAGACTTGGAGT / / AAGGAAGAACC -----------------/ /---------------- GTGAGGC	1.5
	CTGCAG ----------------/ /------------------ CAAAAGGAAA / / TAAGATAATTGTGAGGC	1.1

Total Indel % (Total Large Deletion %) 17.9 (14)

Replicate 3	CTGCAG --/ /---------------------------------- TGTGAGGC	11.2
	CTGCAGACTTGGAGT / / AAGGAAGAACC -----------------/ /---------------- GTGAGGC	1.1
	CTGCAG --/ /---------------------------------- GTGAGGC	0.9
	CTGCAG ----------------/ /------------------ CAAAAGGAAA / / TAAGATAATTGTGAGGC	0.4

b

Total Indel % (Total Large Deletion %) 23.1 (15.6)

T_0-8	CTGCAG --/ /--------------------------------TGTGAGGC	14.1
	CTGCAG ----------------/ /------------------ CAAAAGGAAA / /TAAGATAATTGTGAGGC	0.9

Total Indel % (Total Large Deletion %) 69.6 (62.9)

T_0-9	CTGCAG --/ /------------------------------- TGTGAGGC	62.7
	CTGCAGACTTGGAGT / / AAGGAAGAACC -----------------/ /---------------- GTGAGGC	0.1
	CTGCAG ----------------/ /------------------ CAAAAGGAAA / /TAAGATAATTGTGAGGC	0.0

Total Indel % (Total Large Deletion %) 99.5 (69.4)

T_0-10	CTGCAG --/ /------------------------------- TGTGAGGC	69.4
	CTGCAGCACTTGGAGT / AAGGAAGAACCAAAAGGAAA // TAAGATAATTAGTGAGGC	22.9
