# Supplementary material for: A multiplex guide RNA expression system and its efficacy for plant genome engineering
Source: Plant Methods. 2020 Mar 12;16:37. doi: 10.1186/s13007-020-00580-x (PMC7069183; doi:10.1186/s13007-020-00580-x)
Supplement: Supplementary file 4 — Additional file 4. Small indels induced by pGG-3 in T0 plants. Small indels observed in T0 plants. Wild type (WT) sequences of NaNEC1c are shown with gRNA-binding sequences (underlined) and protospacer adjacent motif (PAM) in red. The DNA sequences of target locus are ranked with the mutation frequency. Indels are presented in blue (insertion) and as dashes (deletion). Total Indel % is the sum of the frequency of small indels and large deletions. The DNA sequences of target locus are ranked with the indel frequency. [file 13007_2020_580_MOESM4_ESM.pdf]

Additional file 4

NaNEC1c

	gRNA3 / gRNA2 / gRNA1	Total Indel %
WT	CTGCAG-ACTTGGAGT // AAGGAAGAACCAAAAGGAAA / / TAAGATAATTGTGAGGC	
	Total Indel %	21.6
T_0-1	CTGCAGAACTTGGAGT // AAGGAAGAACCAAAAGGAAA // TAAGATAATTGTGAGGC	21.2
	Total Indel %	64.7
T_0-2	CTGCAGTACTTGGAGT // AAGGAAGAACCAAAAGGAAA // TAAGATAA- -GTGAGGC	26.4
	CTGCAGTACTTGGAGT // AAGGAAGAACCAAAAGGAAA // TAAGATAATTGTGAGGC	13.2
	CTGCAG-ACTTGGAGT // AAGGAAGAACCAAAAGGAAA // TAAGATAA- -GTGAGGC	8.9
	Total Indel %	96.9
T_0-5	CTGCAGTACTTGGAGT // AAGGAAGAACCAAAAGGAAA // TAAGATAATTGTGAGGC	28.4
	CTGCAGAACTTGGAGT // AAGGAAGAACCAAAAGGAAA // TAAGATAATTGTGAGGC	23.6
	CTGCAGTACTTGGAGT // AAGGAAGAACCAAAAGGAAA // TAAGAT- - - -GTGAGGC	10.5
	Total Indel %	96.2
T_0-7	CTGCAGAACTTGGAGT // AAGGAAGAACCAAAAGGAAA // TAAGATAATTGTGAGGC	44.5
	CTGCAGTACTTGGAGT // AAGGAAGAACCAAAAGGAAA // TAAGATAATTGTGAGGC	19.9
	CTGCAG --/ /--------------------------------- TGTGAGGC	1.5
	Total Indel %	48.2
T_0-11	CTGCAGTACTTGGAGT // AAGGAAGAACCAAAAGGAAA // TAAGATAATTGTGAGGC	25.2
	CTGCAG-ACTTGGAGT // AAGGAA ------------------------/ /----------------------- GAGGC	4.5
	CTGCAGAACTTGGAGT // AAGGAAGAACCAAAAGGAAA // TAAGATAATTGTGAGGC	3.8
