# Supplementary material for: A multiplex guide RNA expression system and its efficacy for plant genome engineering
Source: Plant Methods. 2020 Mar 12;16:37. doi: 10.1186/s13007-020-00580-x (PMC7069183; doi:10.1186/s13007-020-00580-x)

Additional file 5

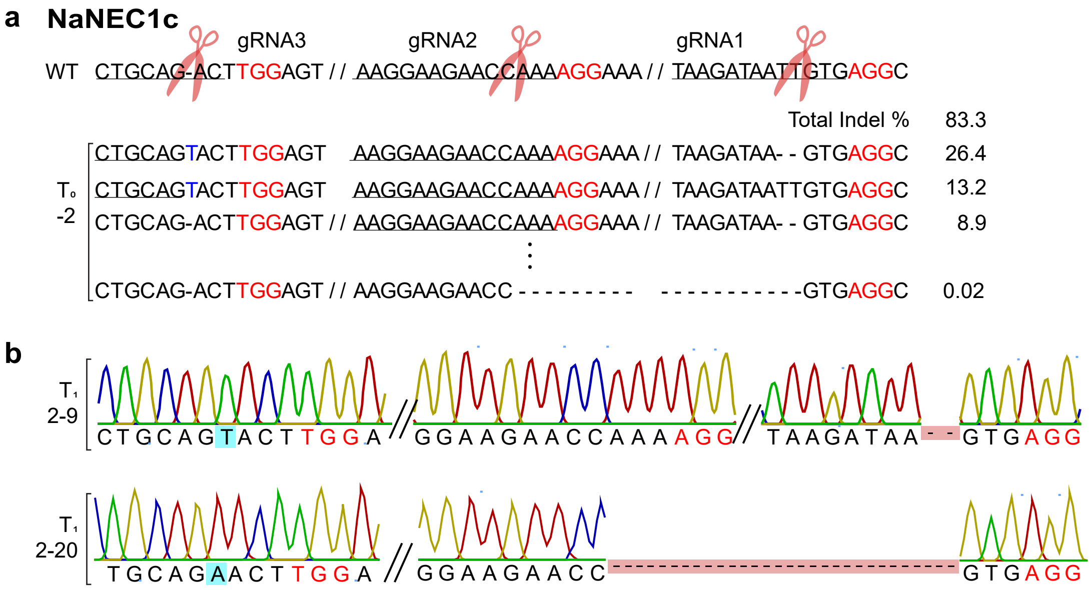

Supplement: Supplementary file 5 — Additional file 5. Heritability of targeted mutations in N. attenuata. (a) Indel mutations observed in T0-2 plants. (b) Sanger sequencing results from two progenies of T0-2 plants. T1-2–9 lines show the small deletion at the gRNA1-cleaved site and the single-nucleotide insertion at the gRNA3-claved site. T1-2–20 lines show the large deletion between the gRNA1- and gRNA2-cleaved sites and the single-nucleotide insertion at the gRNA3-claved site. Total Indel % is the sum of the frequency of small indels and large deletions. The DNA sequences of target locus are ranked with the indel frequency. [file 13007_2020_580_MOESM5_ESM.pdf]
