# Supplementary material for: A multiplex guide RNA expression system and its efficacy for plant genome engineering
Source: Plant Methods. 2020 Mar 12;16:37. doi: 10.1186/s13007-020-00580-x (PMC7069183; doi:10.1186/s13007-020-00580-x)
Supplement: Supplementary file 6 — Additional file 6. Mutation patterns induced by pGG-4 in callus. Large deletions and small indels observed in calli -1, -2, -3, -4, and –5. Wild type (WT) sequences of NaNEC1c and NaEAH1 are shown with guide sequences (underlined) and protospacer adjacent motif (PAM) in red. The DNA sequences of target locus are ranked with the mutation frequency. Indels are presented in blue (insertion) and as dashes (deletion). [file 13007_2020_580_MOESM6_ESM.pdf]

Additional file 6

NaNEC1c

gRNA2 gRNA1

WT CTAGGATCAAAGGAAGAACCAAAAGGAAA / / AACAATGACTAAGATAATTGTGAGGC

Callus	Sequence	Total Indel %
Callus -1		98.4
	CTAGGATCAAAGGAAGAACC --------------------/ /--------------------------------TGTGAGGC	97.6
	CTAGGATCAAAGGAAGAACCAAAAGGAAA / / AACAATGACTAAGATAAT-GTGAGGC	0.5
Callus -2		98.8
	CTAGGATCAAAG ------------------------------/ /---------------------------- ATA --TGTGAGGC	71.3
	---------------------------------/ /------------------------ AACAATGACTAAGATAAT-GTGAGGC	14.6
	---------------------/ /-------------- AAAAGGAAA / / AACAATGACTAAGATAA-- GTGAGGC	10.8
Callus -3		91.3
	CTAGGATCAAAGGAAGAACC --------------------/ /--------------------------------TGTGAGGC	75.1
	CTAGGATCAAAGGA - - - - - - AAAAGGAAA / / AACAATGACTAAGATAATTGTGAGGC	6.3
	CTAGGATCAAAGGAAGAACC ----------------------/ /-------------------------------- GTGAGGC	0.9
Callus - 4		84.8
	CTAGGAT ---/ /---------------------------- AATTGTGAGGC	61.9
	CTAGGATCAAAGGAAGAAC -AAAAGGAAA / / AACAATGACTAAGATAATTGTGAGGC	15.2
	CTAGGATCAAAGGAAGA - -CAAAAGGAAA / / AACAATGACTAAGATAATTGTGAGGC	3.7
Callus -5		88.8
	CTAGGATCAAAGGAAGA ---------------------------/ /----------------------- TAATTGTGAGGC	57.2
	CTAGGATCAAAGGAA ------------------------- / / AACAATGACTAAGATAAT-------/ /-------	21.3
	CTAGGATCAAAGGAAGAAC ---------------- / / AACAATGACTAAGATAATTGTGAGGC	3.1

NaEAH1

gRNA12 gRNA14

WT ACTATCCGTTCAGATTCTTCTTCAGGTAG / / GCATTTGGGCAAGTACTCAAGGGGC

Callus	Sequence	Total Indel %
Callus -1		90.8
	ACTATCCGTTCAGATTCTTCTTCAGGTAG / / CATTTGGGCAAGTACTCAAAGGGGC	32.4
	ACTATCCGTTCAGATTCTTCTTCAGGTAG / / ATTTGGGCAAGTACTCAAAAGGGGC	30.1
	ACTATCCGTTCAGATTCTTCTTCAGGTAG / / CATTTGGGCAAGTACTCTAAGGGGC	21.6
Callus -2		56.9
	ACTATCCGTTCAGATTCTTCTTCAGGTAG / / CATTTGGGCAAGTACTCAAAGGGGC	28.5
	ACTATCCGTTCAGATTCTTCTTCAGGTAG / / CATT--------------------------AAGGGGC	7.8
	ACTATCCGTTCAGATTCTTCTTCAGGTAG / / C--C	7.0
Callus -3		38.4
	ACTATCCGTTCAGATTCTTCTTCAGGTAG / / CATTTGGGCAAGTACTCAAAGGGGC	27.8
	ACTATCCGTTCAGATTCTTCTTCAGGTAG / / CATTTGGGCAAGTACT- - AAGGGGC	5.5
	ACTATCCGTTCAGATTCTTCTTCAGGTAG / / CATTTGGGCAAGTACT - AAGGGGC	1.4
Callus -4		47.4
	ACTATCCGTTCAGATTCTTCTTCAGGTAG / / CATTTGGGCAAGTACTCAAAGGGGC	34.3
	ACTATCCGTTCAGATTCTTCTTCAGGTAG / / ATTTGGGCAAGTACTCAAAAGGGGC	4.0
	ACTATCCGTTCAGATTCTTCTTCAGGTAG / / CATTTGGGCAAGTACT - AAGGGGC	2.4
Callus -5		64.7
	ACTATCCGTTCAGATTCTTCTTCAGGTAG / / CATTTGGGCAAGTACTCAAAGGGGC	44.4
	ACTATCCGTTCAGATTCTTCTTCAGGTAG / / ATTTGGGCAAGTACTCAAAAGGGGC	5.4
	ACTATCCGTTCAGATTCTTCTTCAGGTAG / / CATTTGGGCAAGTACT - AAGGGGC	4.5
